# Supplementary material for: Development of a computer-aided design software for the quantitative evaluation of aesthetic damage
Source: PLoS One. 2019 Dec 18;14(12):e0226322. doi: 10.1371/journal.pone.0226322 (PMC6919621; doi:10.1371/journal.pone.0226322)
Supplement: S1 Code site — 1. (ZIP) [file pone.0226322.s001.zip › [EESC_jr][Projeto]Relatorio_07_21/vendors/select2/docs/_includes/options/data/ajax.html]

## Can Select2 be connected to a remote data source?

Select2 supports connecting to a remote data source using the `ajax` option.

### How can I set the initially selected options when using AJAX?

You can refer to the following Stack Overflow answer if you want to set the initial value for AJAX requests: Select2 4.0.0 initial value with AJAX

### What should the results returned to Select2 look like?

{% include options/not-written.html %}

### Is there a way to modify the response before passing it back to Select2?

You can use the `ajax.processResults` option to modify the data returned from the server before passing it to Select2.

{% highlight js linenos %}
$('select').select2({
ajax: {
url: '/example/api',
processResults: function (data) {
return {
results: data.items
};
}
}
});
{% endhighlight %}

### A request is being triggered on every key stroke, can I delay this?

By default, Select2 will trigger a new AJAX request whenever the user changes their search term. You can set a time limit for debouncing requests using the `ajax.delay` option.

{% highlight js linenos %}
$('select').select2({
ajax: {
url: '/example/api',
delay: 250
}
});
{% endhighlight %}

This will tell Select2 to wait 250 milliseconds before sending the request out to your API.

### How do I tell Select2 which URL to get the results from?

When connecting Select2 to a remote data source, you have the option of using either a single endpoint (a single page which handles all requests) or a dynamic endpoint (one of many pages). You can point Select2 to a single endpoint during initialization by specifying a string for the `ajax.url` option.

{% highlight js linenos %}
$('select').select2({
ajax: {
url: '/path/to/search/endpoint'
}
});
{% endhighlight %}

If there isn't a single url for your search results, or you need to call a function to determine the url to use, you can specify a function for the `ajax.url` option, and this will be used instead. The query parameters will be passed in through the `params` option.

{% highlight js linenos %}
$('select').select2({
ajax: {
url: function (params) {
return '/some/url/' + params.term;
}
}
});
{% endhighlight %}

### I want to add more query parameters to the request, where can this be done?

By default, Select2 will send the query term as well as the pagination data as query parameters in requests. You can override the data that is sent to your API, or change any of the query paramters, by overriding the `ajax.data` option.

{% highlight js linenos %}
$('select').select2({
ajax: {
data: function (params) {
var query = {
search: params.term,
page: params.page
}
// Query paramters will be ?search=[term]&page=[page]
return query;
}
}
});
{% endhighlight %}

### The results that I am seeing never change

Select2 expects that the results that are returned from the remote endpoint are already filtered ahead of time based on the search term. If your remote endpoint just returns the list of all possible options, you may be interested in using Select2's support for data arrays.

### Can an AJAX plugin other than `jQuery.ajax` be used?

Select2 uses the transport method defined in `ajax.transport` to send requests to your API. By default, this transport method is `jQuery.ajax` but this can be changed.

{% highlight js linenos %}
$('select').select2({
ajax: {
transport: function (params, success, failure) {
var request = new AjaxRequest(params.url, params);
request.on('success', success);
request.on('failure', failure);
}
}
});
{% endhighlight %}
